# Cytomegalovirus proctitis mimicking rectal cancer in an immunocompetent elderly patient: a case report

**DOI:** 10.1186/1756-0500-7-799

**Published:** 2014-11-15

**Authors:** Sathyavani Subbarao, Anthony O’Sullivan, Tolu Adesina, Adam M Gwozdz, Julia Rees, Giovanni Satta

**Affiliations:** Department of Pathology, North Middlesex University Hospital, Sterling Way, N181QX London, UK; Elderly Care, North Middlesex University Hospital, Sterling Way, N181QX London, UK

**Keywords:** CMV, Colitis, Cell-mediated immunity

## Abstract

**Background:**

Cytomegalovirus infection is associated with significant morbidity and mortality in immunocompromised patients, but its impact on immunocompetent patients is still poorly understood. Furthermore, there is increasing evidence implying that chronic infection may contribute to a heightened cardiovascular risk.

**Case presentation:**

We describe the case of incidental diagnosis of Cytomegalovirus proctitis in an immune-competent white British elderly gentleman, admitted following a stroke and investigated for rectal cancer following the development of bloody diarrhoea and persistent systemic inflammatory response.

**Conclusion:**

This raised some several interesting points; firstly that we must revise our approach to investigating the immunocompetent elderly patient, secondly, could chronic Cytomegalovirus infection have contributed to the presentation of stroke in this patient and lastly what are the existing evidence for treatment in this population? We use this opportunity to try and address some of these questions and feel that this would be of benefit to the wider audience.

We discuss the risk factors for disease in immune-competent patients and also a brief overview of the benefits of treatment in this population.

## Background

Cytomegalovirus (CMV) infection is a recognised cause of significant morbidity and mortality in immunocompromised patients due to the inability to curb viral replication. The clinical manifestations and duration of symptoms that are seen appear to vary between susceptible groups [1]. Conversely, its impact on immunocompetent patients is still poorly understood. In recent years, the definitions of immunocompetent have been questioned; in this case, we use the term immunocompetent to describe those who are not undergoing chemotherapy or infected with human immunodeficiency virus (HIV). However, we describe an elderly gentleman with multiple co-morbidities whom falls under the spectrum of a broader group of patients who do not have an intact cell-mediated immunity and are susceptible to opportunistic infections [2]. This is becoming an increasing challenge for clinicians who are being faced with such scenarios: a lack of understanding of the pathology of CMV infection and age-related immune changes may lead to a delay in diagnosis and a potential need for treatment. This case highlights the need to be vigilant, especially in those who present with symptoms that are ‘outside the box’.

## Case presentation

We present the case of a 79-year-old white British man on the Acute Stroke unit undergoing Stroke care following a recent left MCA territory Infarct. Past medical history included hypertension, uncontrolled type 2 diabetes and chronic kidney disease stage 3.

During the first few weeks of his admission, his stay was complicated by both an acute NSTEMI with a concomitant troponin-I rise of 4.9 ng/ml (reference levels: 0.0 – 0.04 ng/ml) and systemic inflammatory response. He was initially pyrexial and blood tests revealed a total white cell count (WCC) of 16.5 × 10^9^/L (rising from 12×10^9^/L on admission, neutrophils count 13.0×10^9^/L) and a C-reactive protein (CRP) of 107 mg/L (from 56 mg/L on admission). He was managed conservatively with treatment that included dual anti-platelet therapy.

It was felt that the respiratory system could clinically represent the most likely source of infection although a chest radiogram did not show any consolidation. In view of raised inflammatory markers and ongoing pyrexia, the patient was treated empirically with piperacillin-tazobactam. A full septic screen, including, one set of blood cultures, sputum and urine sample were sent for microbiology culture, which essentially had no significant growth.

**Figure 1:**
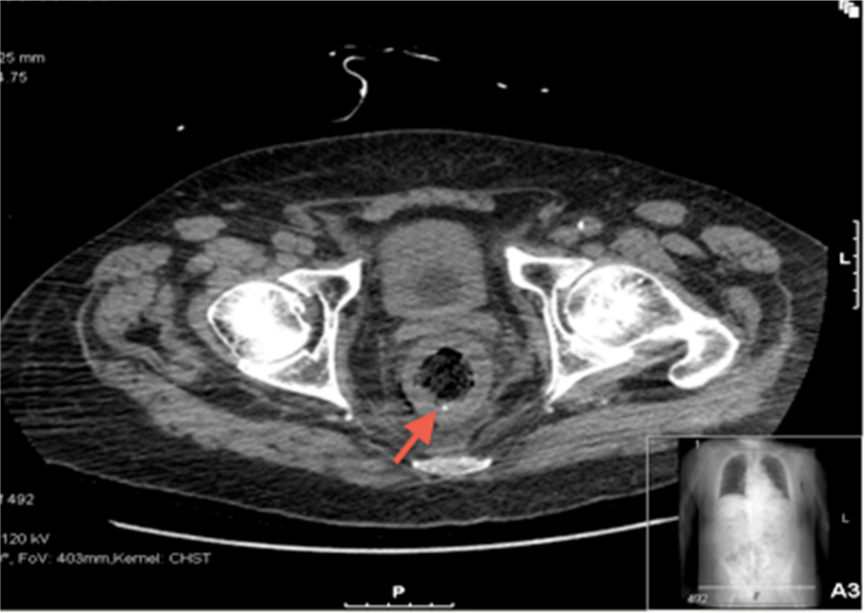
**Computed tomography (CT) image of the lower abdomen.** Red arrow shows concentric wall thickening of the rectum and fat stranding of the perirectal fascia.

His clinical state continued to fluctuate necessitating a change in antibiotic regime to intravenous meropenem, which after 5 days led to an improvement in the patient’s clinical state with associated resolving inflammatory markers.A non-enhanced computed tomography (CT) chest, abdomen and pelvis was performed. Concentric wall thickening of the rectum and fat stranding of the perirectal fascia were the main abnormalities noted with a differential diagnosis of bowel inflammatory process or underlying malignancy (Figure 1).

**Figure 2:**
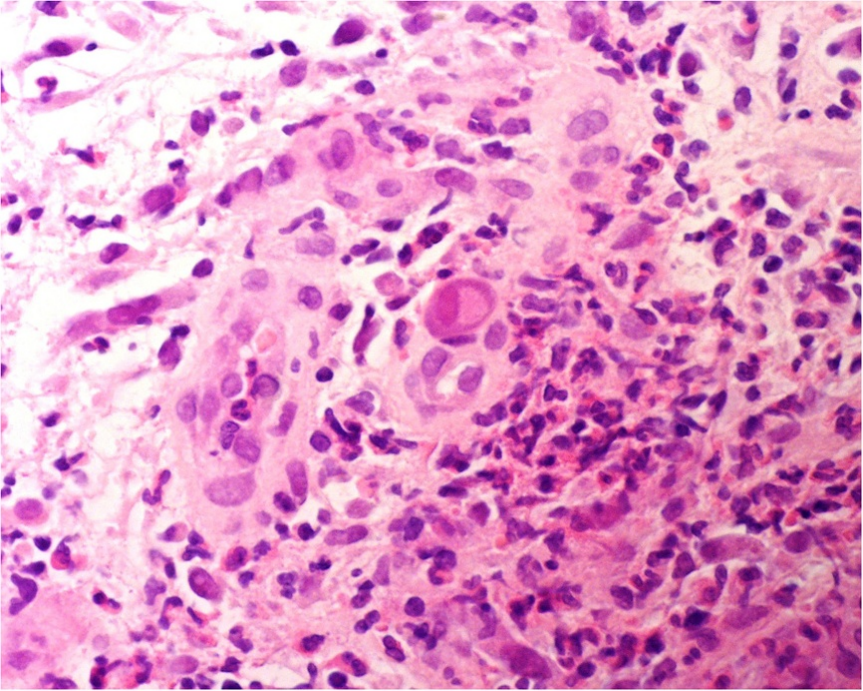
**Haemtoxylin and eosin (H&E) stained slide × 400 showing inflamed granulation tissue containing an enlarged cell with cytomegalovirus inclusion.**

**Figure 3:**
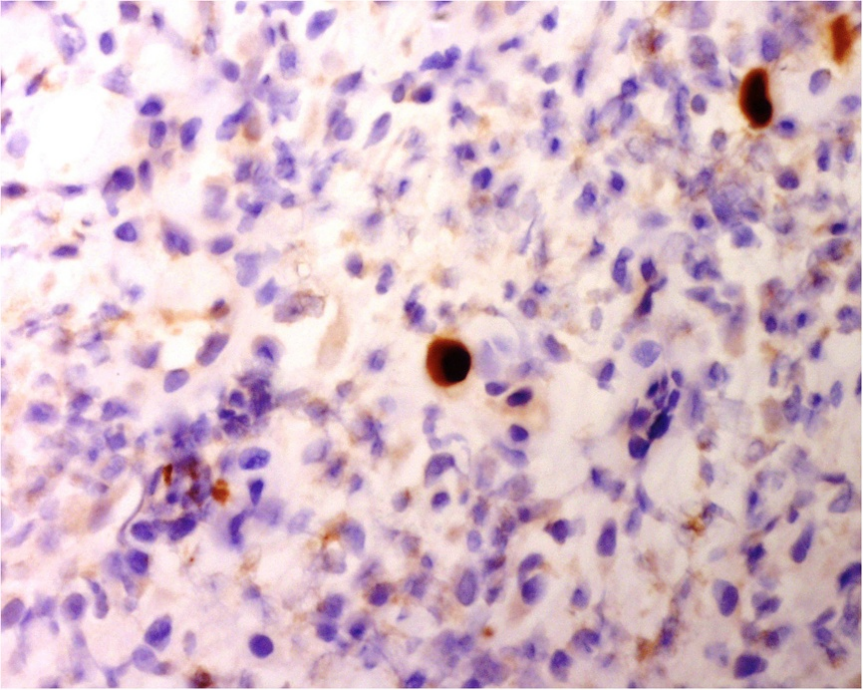
**Immunostaining x 400 for cytomegalovirus shows strong positivity.**

However, he developed diarrhoea (type 7 on the Bristol chart), and intermittent per-rectal bleeding with an associated drop in haemoglobin (Hb) from 12.9 g/dl to 9.3 g/dl. The dual antiplatelet therapy was stopped and multiple stool cultures were sent. Glutamate dehydrogenase (GDH) and *Clostridium difficile* toxins and cultures for enteric community pathogens were all negative. The bleeding was associated with a recurrence of fevers (daily temperatures greater than 38°C) but normal inflammatory markers. Due to the swinging fevers and PR bleeding, attempts were made to directly visualize and biopsy the area of rectal thickening, but it took several days before this was achievable, by which point his fevers and bleeding had once again normalised.Ultimately, flexible sigmoidoscopy revealed what appeared to resemble an ulcerating rectal cancer with a large blind ending malignant cavity. Biopsies were taken and the histological evaluation showed acutely inflamed granulation tissue and ulcer slough, associated with scattered enlarged cells (with eosinophilic nuclear inclusions, Figure 2). Immunostaining was positive for CMV (Figure 3). The histological examination did not confirm any evidence of malignancy. As such, advice and specific tests were sought from the infectious diseases team. CMV viral load was 300 copies/ml; CMV PCR on the fixed tissue sample detected the presence of specific CMV DNA and serological samples showed the presence of CMV IgG and absence of IgM (past infection). An HIV test was negative. The case was discussed in a multidisciplinary meeting where the consensus was that this was CMV proctitis resulting in rectal ulceration.

## Discussion

Cytomegalovirus (CMV) is a double stranded DNA virus, member of the *herpesviridae* family [3]. It is a highly successful human pathogen, which may be, transmitted both vertically and horizontally without an obvious clinical affect on the host. Worldwide, it is estimated that the sero-prevalence of CMV infection is between 40-100% [4], with the largest numbers affecting those from Asia and Africa and high-risk populations. After the initial infection, CMV may persist in a state of latency; effective control of the virus is dependent on an intact CD4+ and CD8+ cell mediated immunity [5]. However, there may still be intermittent asymptomatic viral shedding in both the urine and saliva of both immune-competent and immunocompromised individuals [6].

CMV may manifest itself at the time of the initial infection (primary infection) or at a later stage (secondary), which may be due to reactivation of the latent infection or re-infection via a new exogenous strain [3]. In immunocompromised patients, the inability to control viral replication forms the cornerstone of the devastating disease manifestations that are seen. In contrast, the course run by those with a competent immune system can be more varied. Whilst the majority of those affected may be asymptomatic or present with a generalized mononucleosis-like syndrome, other clinical manifestations are not as infrequent as previously thought [2]. The gastrointestinal tract is the most common site affected by CMV in immune-competent patients with a predilection for the colon and rectum [2]. However, the term *immunocompetent* could be considered a misnomer in certain situations. Previous studies have shown that CMV colitis spontaneously resolves in patients <55 years [7]. Conversely, specific groups such as diabetics, age ≥55 years, chronic renal failure, pregnancy and untreated non-haematological malignancy appear to adversely influence survival [7]. It is known that all these conditions do have a modulatory affect on the immune system; for example, both aging and diabetes are associated with a decline in cell-mediated immunity and a dysregulation in the cytokine responses, which is reflected by the increase in the numbers of overall infections [8]. In fact, age-associated dysregulation in the immunity (Immune-senescence) is becoming increasingly well characterised. Aging is associated with reduced pool of naïve T cells and CMV infection further compounds this, through expansion of CMV specific CD8+ and CD4+ memory T cells [9]. Moreover, observational data shows that CMV sero-positivity increases the risk of cardiovascular death in individuals over the age of 65 years [10]. Thus, whilst these groups of patients are not immunocompromised in the traditional sense, they certainly do not have a preserved immunity and should be investigated as such. As a testimony to this, our patient was indeed afflicted by a number of co-morbidities that would be altering his immune responses: advanced age, uncontrolled type 2 diabetes and renal impairment. If this gentleman was infected by the human immune-deficiency virus (HIV) and had presented with per-rectal bleeding, he would have had a CMV PCR performed immediately as part of routine investigations, especially in view of the abnormalities seen on the CT scan.

In this case, the diagnosis of CMV infection was an incidental finding, based on the presence of intranuclear inclusion bodies at the histological examination. These inclusions are known as *owl’s eye bodies*, which stain dark pink on haematoxylin and eosin stain [11]. We also demonstrated the molecular amplification of the viral DNA within the tissue and a low level viraemia was seen several weeks after the onset of symptoms. We can speculate that in this case, CMV proctitis was due to a re-activation of a latent infection evidenced by the presence of IgG antibodies and absence of IgM. Whether the initial presentation arose as a consequence of systemic sepsis or whether there was a high CMV viraemia at the start of admission driving the sepsis like syndrome remains to be elucidated. The cytokine effects of sepsis driving CMV viraemia have previously been described; it is appears that the presence of pro-inflammatory cytokines, tumour necrosis factor-alpha (TNF-alpha) and interleukin (IL)-1 beta in the early stages of sepsis reactivate CMV from latency [12]. Furthermore, the later stages of sepsis are characterized by immunosuppressant interleukins such as IL-10 and IL-4 [13]. These are believed to be involved in CMV proliferation following reactivation. However, several courses of broad-spectrum antibiotics failed to completely control the systemic inflammatory response in this patient, suggesting that this might have been driven by a large CMV viraemia. A recent systematic review identifying the contribution of CMV to outcomes in the critical care unit showed that CMV infection occurred in 0-36% of critically ill patients and the risk of CMV infection was five-fold higher in those patients presenting with sepsis [14]. A further question arising from this case is whether CMV itself was an independent driver of the stroke and myocardial infarction, rather than just a bystander. Several studies previously, have shown that uncontrolled viraemia in HIV-infected patients have a higher cardiovascular risk compared to patients with undetectable viral loads on anti-retroviral therapy [15]. Similarly, in vitro studies have demonstrated that CMV directly invades endothelial cells and has potent procoagulant properties [16]. There is no current causal link between CMV seroprevalence and atheroma formation. However, epidemiological evidence shows accelerated atherosclerosis in the presence of CMV in patients following cardiac transplantation [17].

By the time the diagnosis of CMV proctitis was reached, patient’s symptoms had subsided, therefore obviating the need for treatment. However, there are no randomized controlled trials examining the role for antivirals in this population, with limited evidence available. Whilst the incidence of CMV-related disease in the immunocompetent population may be increasing, many recover without intervention. It would therefore be impractical to conduct a large randomized trial establishing clinical efficacy and toxicity. We therefore advocate the use of antivirals in such patients on a named basis, balancing the risks and benefits of treatment.

## Conclusion

This case highlights an unusual clinical presentation of CMV proctitis in a patient whom rectal cancer was initially top of the differential diagnosis. The endoscopic and radiological findings, which were finally attributed to CMV infection, mimicked a malignancy. Whilst exclusion of a cancerous lesion was clearly a priority, this lead to a delay in the diagnosis of the CMV infection. Moreover, the persistent systemic inflammatory response was likely to have been secondary to a chronic CMV viraemia rather than an overt bacterial sepsis; additionally we pose the question of whether an uncontrolled viraemia could have contributed to the initial stroke. We therefore suggest that a high index of suspicion is important for an early diagnosis and potential instigation of treatment.

## Consent

Written informed consent was obtained from the patient’s next of kin (as the patient was elderly and neurologically impaired) for publication of this Case Report and any accompanying images. A copy of the written consent is available for review by the Editor-in-Chief of this journal.
